# Curriculum learning-based strategy for low-density archaeological mound detection from historical maps in India and Pakistan

**DOI:** 10.1038/s41598-023-38190-x

**Published:** 2023-07-12

**Authors:** Iban Berganzo-Besga, Hector A. Orengo, Felipe Lumbreras, Aftab Alam, Rosie Campbell, Petrus J. Gerrits, Jonas Gregorio de Souza, Afifa Khan, María Suárez-Moreno, Jack Tomaney, Rebecca C. Roberts, Cameron A. Petrie

**Affiliations:** 1 Landscape Archaeology Research Group (GIAP), Catalan Institute of Classical Archaeology (ICAC), Pl. Rovellat s/n, 43003 Tarragona, Spain; 2 Catalan Institution for Research and Advanced Studies (ICREA), Passeig Lluís Companys 23, 08010 Barcelona, Spain; 3 Computer Science Department, Computer Vision Center, Universitat Autònoma de Barcelona, Edifici O, Campus UAB, 08193 Bellaterra, Spain; 4 Banaras Hindu University, Ajagara, Varanasi, Uttar Pradesh 221005 India; 5 McDonald Institute for Archaeological Research, University of Cambridge, Downing St., Cambridge, CB2 3ER UK; 6 Complexity and Socio-Ecological Dynamics (CaSEs) Research Group, Universitat Pompeu Fabra, Barcelona, Spain; 7 Department of Archaeology, University of Cambridge, Downing St., Cambridge, CB2 3DZ UK

**Keywords:** Computational science, Scientific data, Environmental impact, Ecological modelling, Population dynamics

## Abstract

This paper presents two algorithms for the large-scale automatic detection and instance segmentation of potential archaeological mounds on historical maps. Historical maps present a unique source of information for the reconstruction of ancient landscapes. The last 100 years have seen unprecedented landscape modifications with the introduction and large-scale implementation of mechanised agriculture, channel-based irrigation schemes, and urban expansion to name but a few. Historical maps offer a window onto disappearing landscapes where many historical and archaeological elements that no longer exist today are depicted. The algorithms focus on the detection and shape extraction of mound features with high probability of being archaeological settlements, mounds being one of the most commonly documented archaeological features to be found in the Survey of India historical map series, although not necessarily recognised as such at the time of surveying. Mound features with high archaeological potential are most commonly depicted through hachures or contour-equivalent form-lines, therefore, an algorithm has been designed to detect each of those features. Our proposed approach addresses two of the most common issues in archaeological automated survey, the low-density of archaeological features to be detected, and the small amount of training data available. It has been applied to all types of maps available of the historic 1″ to 1-mile series, thus increasing the complexity of the detection. Moreover, the inclusion of synthetic data, along with a Curriculum Learning strategy, has allowed the algorithm to better understand what the mound features look like. Likewise, a series of filters based on topographic setting, form, and size have been applied to improve the accuracy of the models. The resulting algorithms have a recall value of 52.61% and a precision of 82.31% for the hachure mounds, and a recall value of 70.80% and a precision of 70.29% for the form-line mounds, which allowed the detection of nearly 6000 mound features over an area of 470,500 km^2^, the largest such approach to have ever been applied. If we restrict our focus to the maps most similar to those used in the algorithm training, we reach recall values greater than 60% and precision values greater than 90%. This approach has shown the potential to implement an adaptive algorithm that allows, after a small amount of retraining with data detected from a new map, a better general mound feature detection in the same map.

## Introduction

The past 100 years and, in particular, the second half of the twentieth century, have seen extensive urban growth and the large-scale implementation of mechanised agriculture and irrigated systems in India and Pakistan, causing irreversible effects on the landscape. Among other lasting impacts, such as the implementation of large-scale irrigation systems, river avulsion and flooding, there have been much systematic flattening, for cultivation and construction, of hundreds, if not thousands, of archaeological settlement mounds^1–3^. These archaeological mounds with their distinct elevation, colour and form are an indicative feature of past settlements and anthropogenic modifications of the landscape. Given their partial or total destruction, these are no longer detectable by other types of sources such as LIDAR or satellite imagery^4,5^. Historical maps are therefore often the only source of information about the location and size of those lost sites. Available satellite images of the Indian subcontinent date back to 1972 thanks to the Landsat satellite programme^6^, but detailed mapping of this region through triangulation dates back to 1802 and the start of the Great Trigonometrical Survey. Later, during the period of British rule in India and Pakistan (1858–1947), the Survey of India (SoI) continued the systematic mapping of the whole subcontinent.

**Figure 1:**
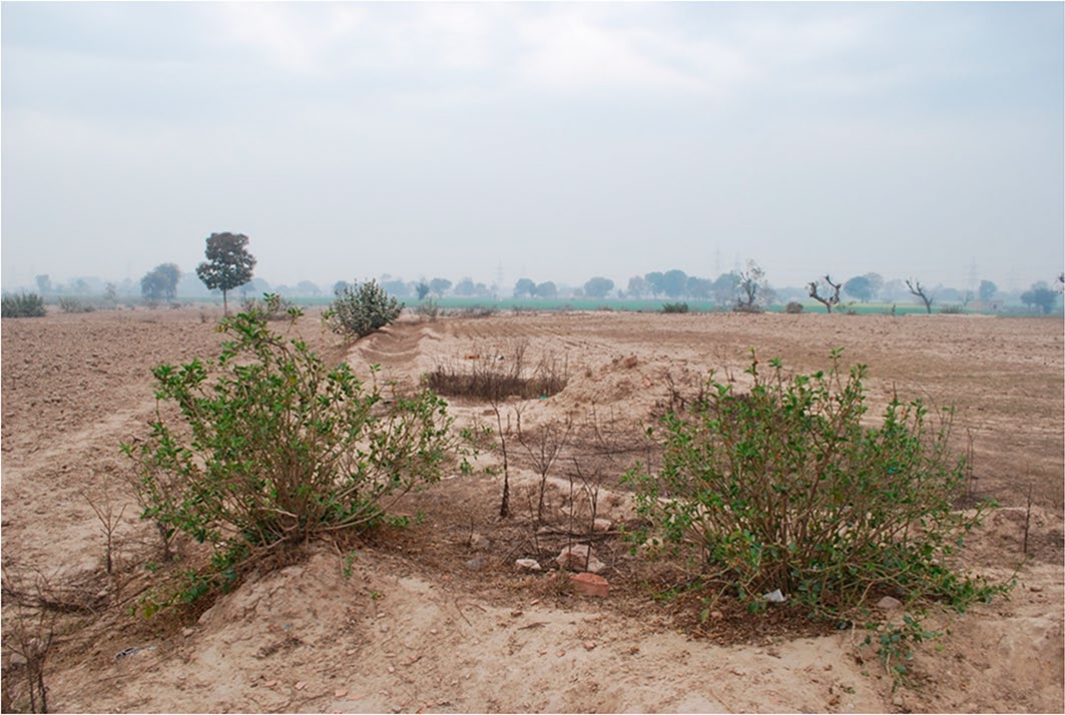
Archaeological remains found where the historical maps indicated mounds. View from an elevated mound feature in northwest India (L742). Image from Green et al. ^3^, Fig. 2. Reproduced here under the terms of the CC-BY 4.0 license in which it was originally published.

The SoI maps were originally intended to be geographic maps and depicted different topographic features including mound features, many of which, as further research has shown^3^, are in fact archaeological sites (Fig. 1). It is impossible to calculate the percentage of mounded sites that were not drawn in the SoI maps, given the disappearance of sites during the last 100 years and the lack of reliable large-scale archaeological survey data. However, all sites listed as being protected at the time the map surveys took place are indicated on the historic maps, including sites like Harappa and Taxila^7^. Also, many major sites that were documented on the map sheets were not ‘discovered’ by archaeologists for many years if not decades, including the major Indus Civilisation city sites of Mohenjo-daro, Rakhigarhi and Dholavira. Furthermore, ground truthing has revealed there is a correlation between these mound features and proto-historical and historical sites dating to various periods from the period of the Indus Civilization onward^3^.

Deep Learning (DL) has been widely used in recent years to aid archaeological survey by using different resources such as lidar data^4–6,8^ and drone imagery^9^. This study continues the work carried out by several authors for the detection of archaeological sites using historical maps^1–3^. Previous studies made by Garcia-Molsosa et al. focused on the present district of Multan in the Pakistani province of Punjab. The series of maps used in this study had similar production standards^10^. Although this previous approach produced satisfactory results it presented some drawbacks:

1. It employed a reduced series of maps of similar chronologies, depiction standards, scanning quality and preservation. This ideal situation, however, proved not to be the norm when a much larger collection of maps was assembled. The larger collection presented important variations in coloration, representation standards, scanning quality and preservation, which enormously complicated the large-scale application of these initial detectors^10^ and significantly reduced their detection capabilities.
2. The initial algorithms were designed in a proprietary web-based geospatial machine learning (ML) platform. The models were not available for download, analysis or free distribution and the processing was expensive, prohibitively so when considering large areas such as the one under investigation.

**Figure 2:**
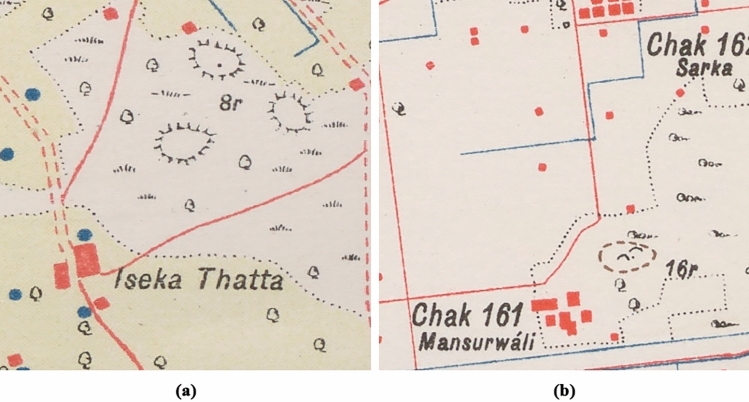
The two types of mound features depictions that need to be detected in historical maps: (**a**) hachure [8r], and (**b**) form-line [16r].

The study presented in this paper uses the historical maps produced in the late nineteenth and early twentieth century by the SoI with the aim of detecting two of the most common ways of drawing mound features (hachure and form-line, see Section "Deep learning model" for further details), which are similar to those depicted by the French in Syria and Lebanon^10^ (Fig. 2). Our research seeks to develop two DL segmentation algorithms for mound feature detection, one for each mound type, extending the detection to an area of 470,500 km^2^ (most of which corresponds to the Indus River Basin), the largest area in which such an approach has ever been applied^4^, and to all types of maps, thus increasing the complexity of the analysis. We have employed a Region-based Convolutional Neural Network (R-CNN) segmentation algorithm as it collects information about not only the location of the mound feature, but also about its shape and extent.

Automated detection processes require large amounts of data for their training (typically in the order of tens of thousands of individual examples), but this is not common in archaeology where the number of known archaeological samples to train a ML algorithm is very low, as in this case study. Other studies with similar elements such as burial mounds^4^, have shown that despite having limited training data, features of interest are detectable due to the characteristic circular shape of the tumuli, which presented few variations. The archaeological elements of this study, despite being mound features like those of previous studies where we encountered a similar problem, are much more diverse. Since they are symbols drawn by human hands and not images of their actual form, whether aerial or satellite, the features are noticeably divergent in style from each other. Consequently, a relatively small amount of training data was not enough to achieve meaningful results.

In computational archaeology, trained ML models have been shown to perform worse in areas with low-density of archaeological features than in high-density ones (e.g.^11,12^). When performing large-scale detection with few sites, many False Positives (FPs) are introduced (typically many more than the True Positives (TPs)), which severely reduces the accuracy of the algorithm. However, real archaeological scenarios typically present low-densities of archaeological sites that need to be detected, at least compared to other typical objects in Computer Vision studies (such as cars, trees, buildings, ships, etc.). During a survey, the actual density of archaeological features is unknown, so to be a useful tool, the developed ML algorithm must also provide good results for low-density areas.

Therefore, the use of ML approaches in archaeology entails a series of idiosyncratic challenges: including the customary small amount of archaeological data for training and the usual low-density of archaeological features. In this article we will implement a series of data augmentation (DA) techniques and learning strategies to resolve these two issues.

The main goal of this article, besides the successful detection of mound features within acceptable parameters of precision and recall, will address these two issues by designing a workflow for the correct detection of archaeological features (1) in low-density areas and (2) with little amount of training data.

## Materials and methods

In this study, a total of 645 maps, provided by the Cambridge University Library and the British Library have been used. These historical maps were produced and distributed by the SoI, and can be classified into different periods characterized by the then current surveyor general of the SoI, including C. Straham (1898–1899), G.C. Gore (1900–1902), F.B. Longe (1904–1907), and S.G. Burrard (1912–1913). Maps produced under A.R. Quraishi (1954) in his role of Surveyor General of the survey of Pakistan have also been included.

### Map digitisation and georeferencing

**Figure 3:**
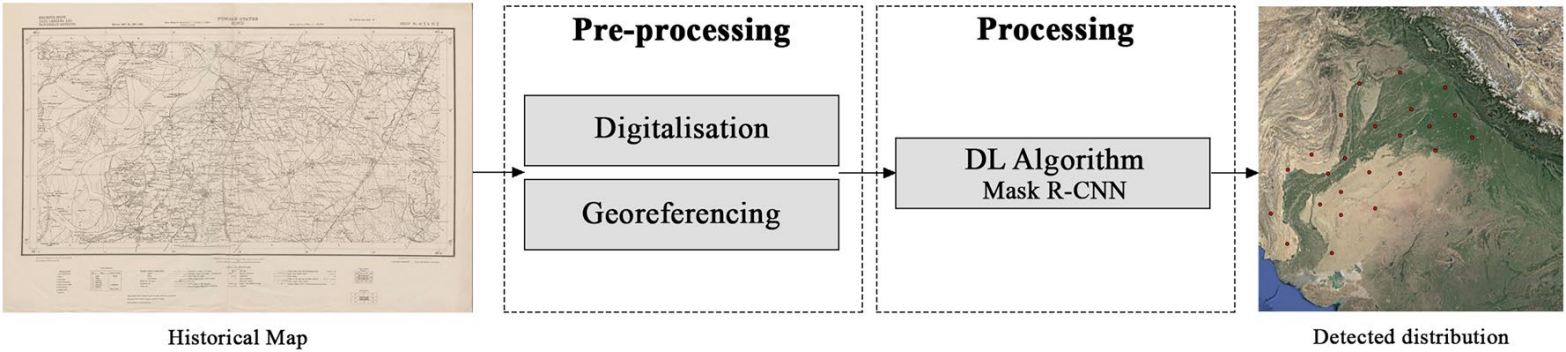
Scheme of the workflow for the detection of mounds in historical maps.

Before proceeding with the training of the DL algorithm, all 645 maps used for this study had to be scanned and georeferenced (Fig. 3). The scanning process was done by different institutions and individuals, in different periods and using different means and resolutions as a result of the different histories, means, and the procedures of the different institutions hosting and scanning them. After the digitalisation of the maps, they were georeferenced using a minimum of 12 Ground Control Points (GCPs) and an average of 25, geometrically distributed within the map to achieve a good distribution and an accurate transformation. The GCPs were obtained from georeferenced high resolution RGB satellite imagery available as Web Map Services layers in QGIS software (several versions were employed)^13^. The georeferencing process mainly used second order polynomials, which was the preferred method, and was applied to most maps. On few occasions, when the maps had suffered lineal distortions due to folds in the map surface, the adjust transformation was used. These methods produced average Root Mean Square Error (RMSE) values of 0.00035° (ca. 33.7–38.8 m at this latitude) using a second order polynomial and 0.00010236° (ca. 10.3 m with a maximum value of ca. 26.8 m) using the adjust transformation. Since the mounds under consideration are typically much larger than these values, the georeferencing process results in mound feature locations, which, largely overlap the real locations (for more details on the georeferencing process see^1^).

### Deep learning model

In recent years, R-CNN models have become very common in archaeological survey, highlighting segmentation algorithms such as mask R-CNN^9^ and DeepLabV3+^14^. For this study, we developed two mound symbol detection DL algorithms using mask R-CNN^15^, since we are looking for instance segmentation rather than semantic. Mask R-CNN detects objects in an image while simultaneously generating a high-quality segmentation mask for each instance^16^. It extends Faster R-CNN^17,18^ by adding a branch for predicting segmentation masks, a small fully convolutional network (FCN)^19^, on each region of interest (RoI), in parallel with the existing branch for classification and bounding box regression. Mask R-CNN is simple to train and adds only a small overhead to Faster R-CNN^16^. Likewise, VGG Image Annotator (VIA) from the University of Oxford has been used to label mound features^20^.

The digitized and georeferenced historical maps are 3-channel RGB images and we have cropped them into 512 × 512 pixel images to save computing costs. Of the 645 maps used, only 43 contained known mound features, which have been used for training and validation: 286 hachure and 103 form-line mound features. Of those maps, 22 were used for training, including 168 hachure and 26 form-line mound features, and 21 were used for validation, including 118 hachure and 77 form-line mound features. In addition, given the small number of known mound features, another 21 maps, chosen randomly from the 645 original maps, were manually analysed. In this way, we have been able to create another dataset, the test dataset with 230 hachure and 137 form-line mound features, to evaluate the model obtained from training and validation for a second time.

**Figure 4:**
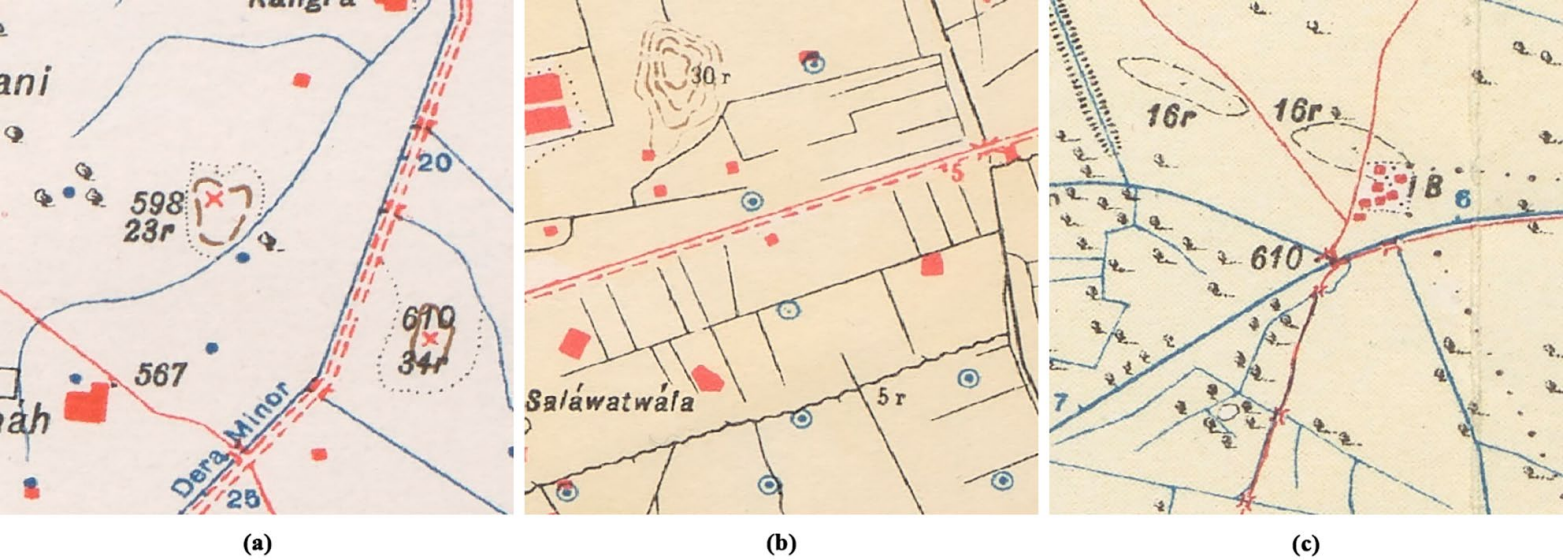
Different form-line typologies found in historical SoI maps: (**a**) dashed [23r] and solid line [34r] mound feature, (**b**) mound feature with concentric lines [30r], and (**c**) road-like black line mound feature [16r].

SoI map styles, colours and symbology depended on the date the maps were produced, the team drawing them, the region and the print quality of the map^1^. Each map type also corresponds to a drawing style and, therefore, to a different mound colour, despite corresponding to the same type of mound feature. There are three typologies by which mound features are represented in the SoI maps, of which the most common ones are the hachure and the form-line mound feature. The hachure is depicted with many fragmented lines which show the orientation of the slope, whereas the form-line mound features are drawn to represent one elevation (Fig. 2). The third type of mound feature representation on SoI historical maps is shaded-relief. Although these are also present on the maps under study, they are not included in the automated detection given the low correspondence of this type of mound feature with archaeological sites, where 86.36% of the examples visited on the ground were found not to be archaeological sites^3^. We have focused the form-line algorithm on detecting only its most common typology, as opposed to the hachure algorithm which detects all types of hachure depiction. This is due to the fact that other form-line mound feature types (mound feature with concentric lines, with continuous line and black ones) do not have their characteristic shape and they are similar to other typologies that have no relation to archaeological features, such as road and slope lines (Fig. 4). Likewise, cropped mound features by the process of clipping maps to 512 × 512 pixels have not been detected because there are form-line and hachure-shaped features that are not closed in a circle and are not mound features.

ML algorithms like Mask R-CNN typically evaluate their models on images that contain labelled objects and do not evaluate those without labels. Since our goal is to demonstrate the good performance of the model in low-density areas, we have created artificial mound labels on all those images without real mounds to force the analysis in them. This way, the algorithm also evaluates the presence or absence of mounds in areas of the map where we know there are no mound features to better assess its precision. The 4 × 4 pixel artificial mound features are placed in the upper left corner of the images and will never be detected, as our algorithm discards any detection at the edges of the images (10 pixels from the edge) to avoid FPs derived from cropped symbols. These artificial mound features will never be detected, but these areas will be analysed, allowing our model to analyse both high-density and low-density areas.

If our study had focused on areas with a high-density of mound features, our method and research could have ended here since we obtained good results after the first training for both hachure and form-line mound features. However, the majority of archaeological surveys are conducted in areas with a low-density of sites, or in places where the density of archaeological features is undetermined. Therefore, if we have looked at the reality of archaeological research and analyse the results of the first training for low-density areas, we observe that it is necessary to refine the model given the high number of FPs present in the results.

### Model refinement

The high number of FPs present in the first training was due to the limited number of training data available. Therefore, with the idea of introducing new training data, both positive and negative, various DA techniques have been applied. The first DA methods developed were mound feature random translation (DA1), random rotation (DA2), and the so-called Doppelgänger technique (DA3).

**Figure 5:**
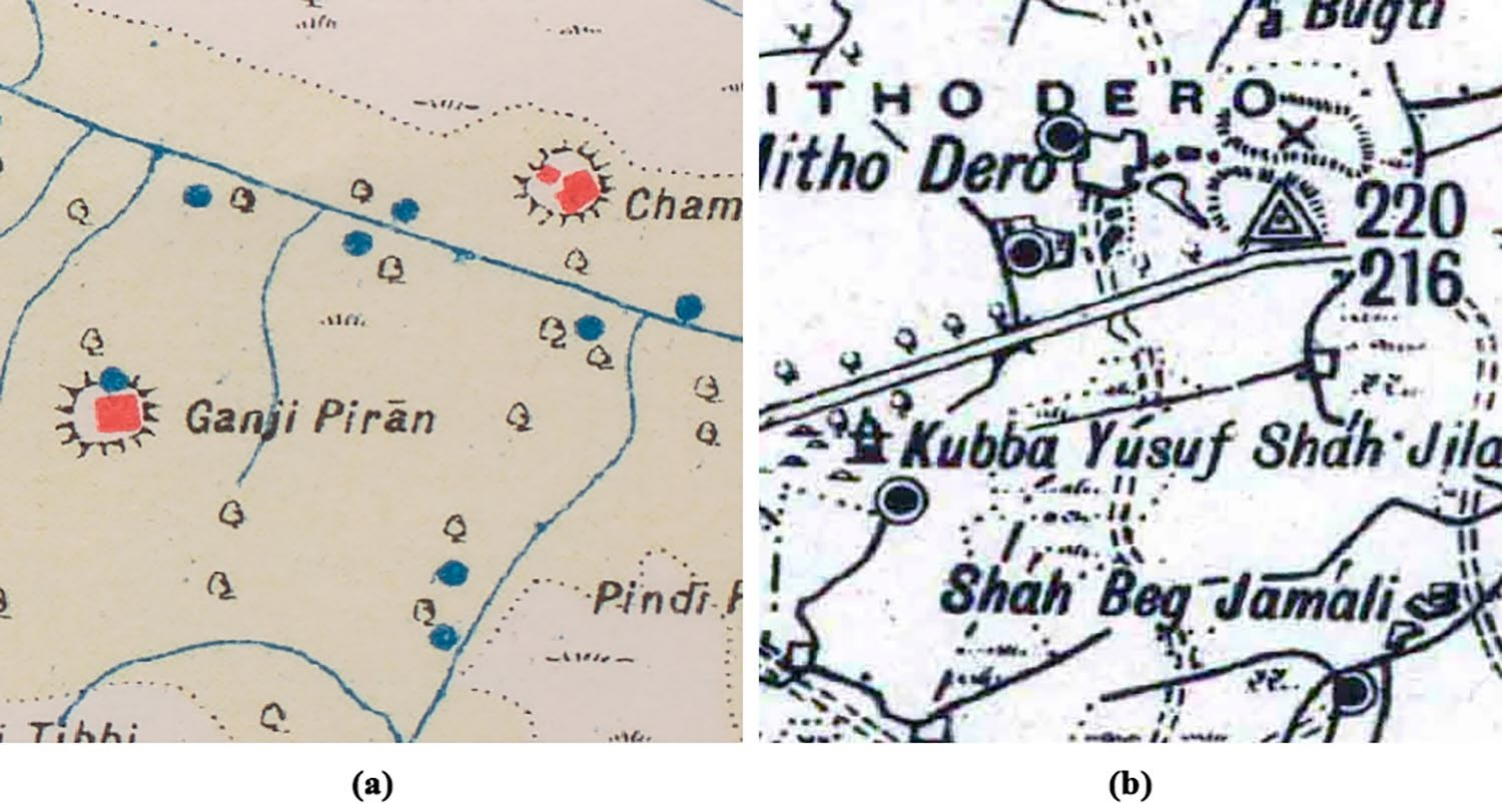
Some examples of hachure mound features containing different symbols inside, as well as two types of map background colour (**a** and **b**).

For each type of mound feature and algorithm, 1500 new artificial mound features were used, created randomly from the original ones used for training, and they were placed, by an automated process, randomly on all the maps used in training, implementing both DA1 and DA2. When pasting these artificial mound features at random on each of the training maps, they were emptied of any other feature than the actual mound depiction as they contained various symbols unrelated to the mound feature itself, thus avoiding possible FPs derived from the presence of these symbols, but also because the training maps had different background colours and the inclusion of these features would have created artificial colour-related features (Fig. 5).

**Figure 6:**
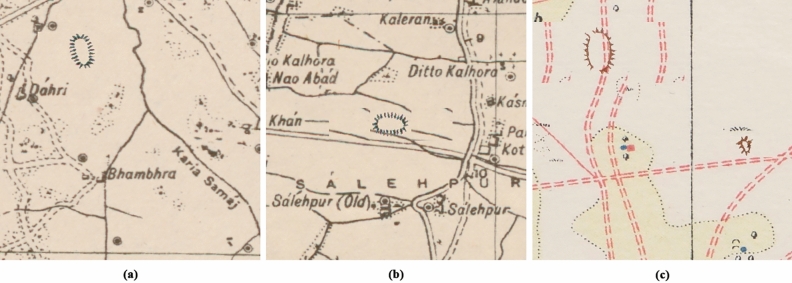
First DA techniques used: (**a**) random translation (DA1), (**b**) random rotation (DA2), and (**c**) the so-called Doppelgänger technique (DA3).

In order to avoid FPs due to common symbols on the maps such as roads, grass and trees where these new artificial mound features could have been placed randomly, DA3 was developed to copy the inside of each mound feature and to paste it to the outside of the mound feature so that it can be taken as negative training and just the mound feature as positive data (Fig. 6). In this study it has been decided not to implement other possible DA techniques such as resizing, because mound features of different sizes are drawn differently than the resized mound feature itself. The hachure and form-line shapes are different for each size, increasing or decreasing the number of strokes drawn. Therefore, noise would be introduced into the algorithm. The entire DA process has been done using our own script written in Python (see Data availability Section for further details).

**Figure 7:**
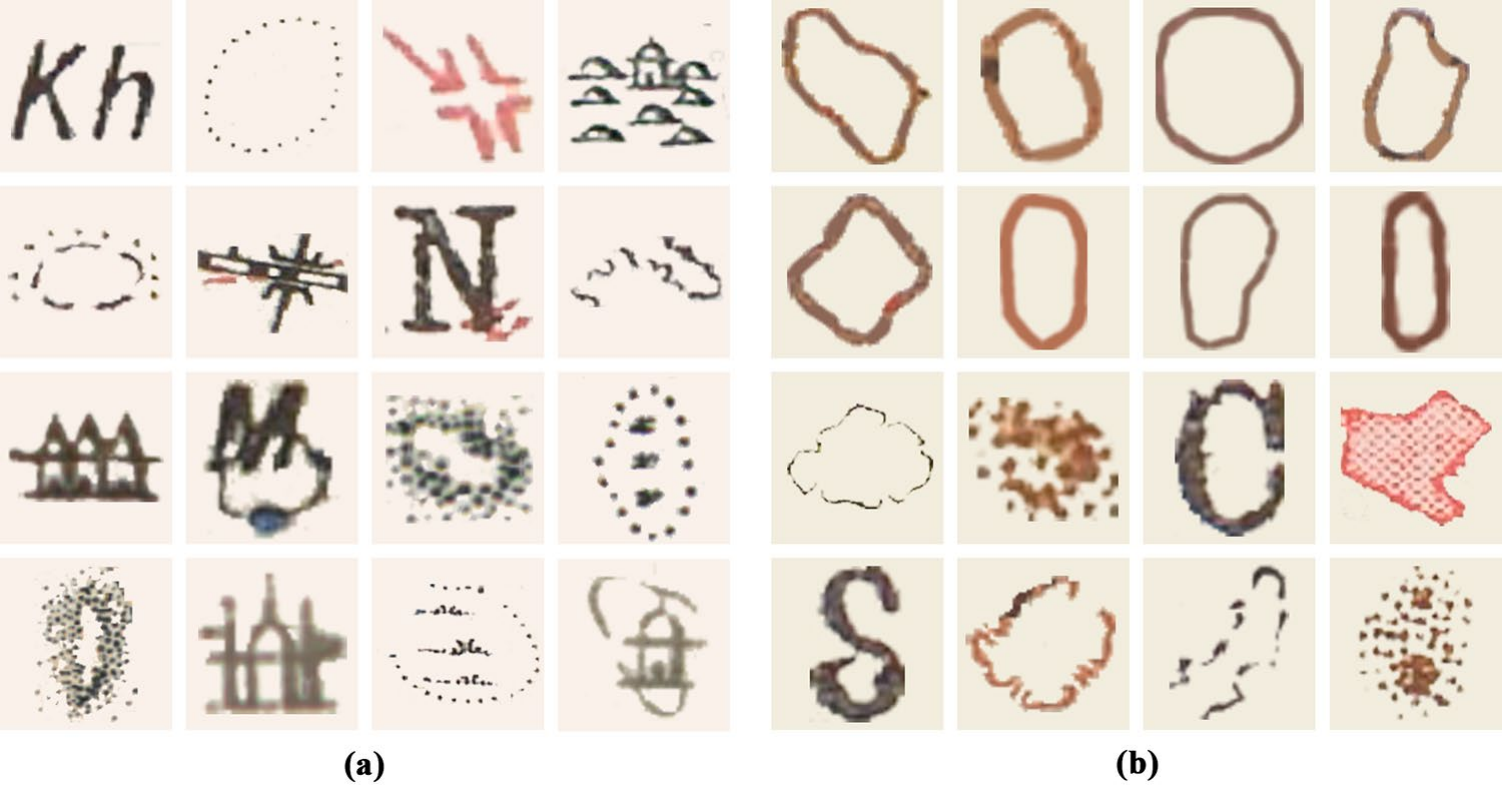
Some FPs used as negative training data for refinement (DA4): (**a**) hachure FPs and (**b**) form-line FPs.

After increasing the positive and negative training, the number of FPs detected was considerably reduced, but a series of specific FPs was still maintained. In order to further reduce these, a refinement stage (DA4) was included (Fig. 7). In both cases the same correct mound features were used as continuous line circles for negative training data, so the algorithm could decide that continuous lines are not mound features. The total number of elements used as refinement is 88 for the form-line and 127 for the hachure ones, which have been placed using the DA1 and DA2 techniques up to a total of 8800 for the form-line algorithm and 12,700 for the hachure model.

### Curriculum learning approach

**Figure 8:**
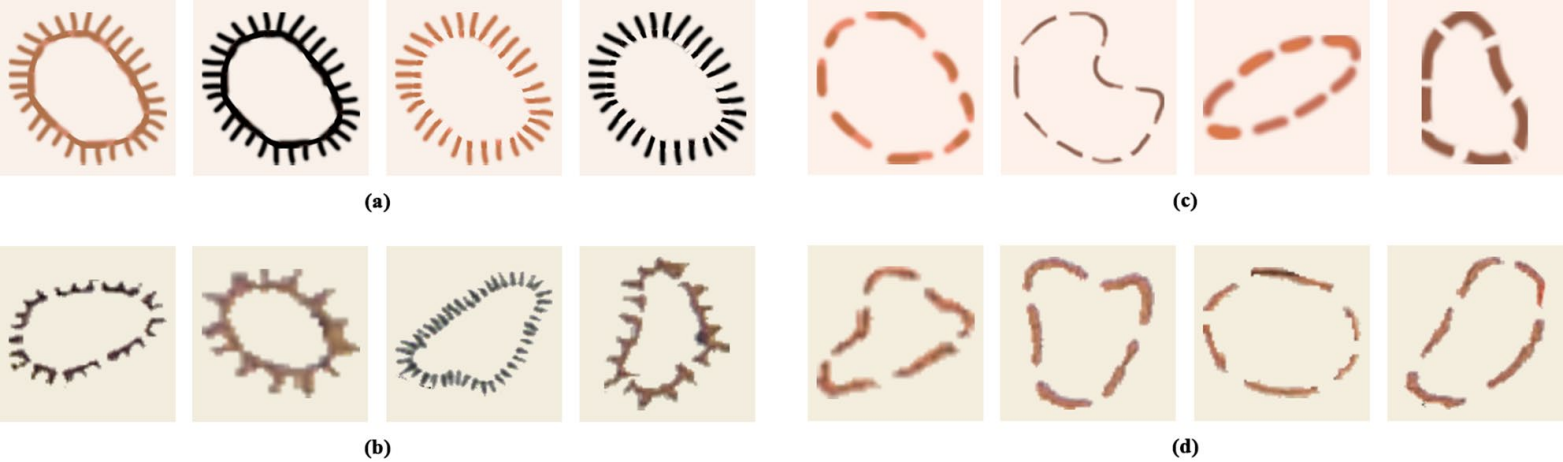
Hachure and form-line mound feature datasets for CL: (**a**) examples of synthetic hachure mound features (DA5), (**b**) examples of original hachure mound features, (**c**) examples of synthetic form-line mound features (DA5), and (**d**) examples of original form-line mound features. The synthetic data (**a** and **c**) is for the first training of each of the two algorithms and the original data (**b** and **d**) for the second training also for both algorithms.

Thanks to these DA methods we managed to reduce the number of FPs considerably, increasing the precision of the model. However, we stopped detecting some of the mound features that were initially detected, which also reduced the recall value. For this reason and with the aim of improving the accuracy metrics, it was decided to implement a Curriculum Learning (CL) strategy with synthetic data (DA5) (Fig. 8).

**Figure 9:**
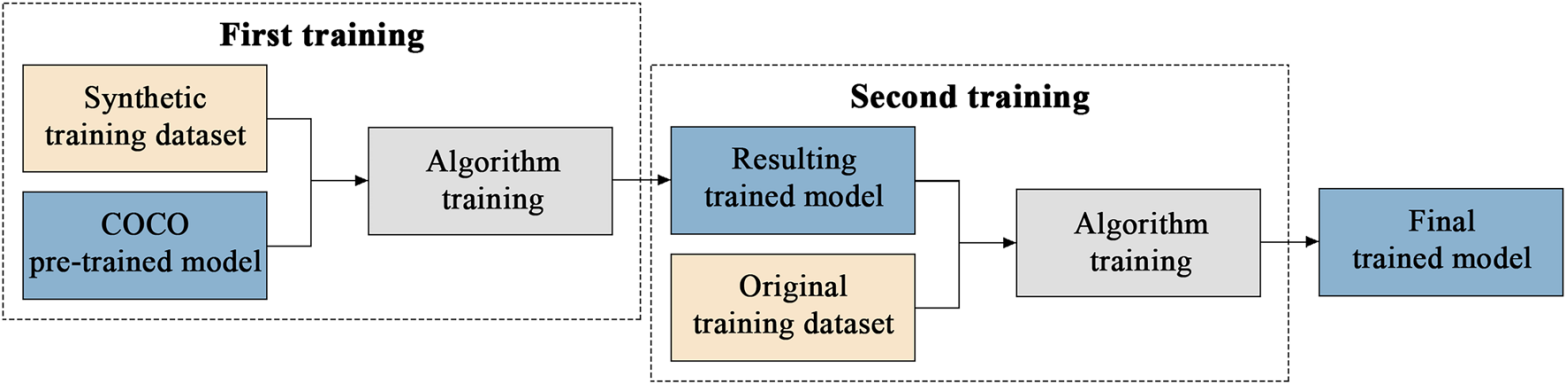
CL process scheme where stages with more complex aspects of the mound features are gradually included: first the synthetic dataset with DA and second the original with DA.

Firstly, CL is a way to gradually introduce complexity to the model through more training phases^21^. Secondly, the lack of data forced us to create synthetic data for each mound feature class (DA5), which we have used to make the algorithm learn through a CL strategy. In this way, the algorithm first learns the basics from the synthetic data and then more complex variations from the few known mound features in its second training, as a fine-tuning stage (Fig. 9). A total of 75 synthetic mound features were created for each of the two types.

### Model filtering

Previous ground-truthing studies in India^3^, which included only a small number of well-preserved archaeological mounds, showed that those mound features smaller than 200 m in diameter were mostly not archaeological sites, with hachure features adjacent to villages often corresponding to ponds or upcast from the creation of those ponds. Only 7.96% of the hachure and 25.83% of the form-line mound features of less than 200 m corresponded to archaeological sites^3^. Likewise, research on mound features in Pakistan showed that many of the small mound features less than 100 m in diameter were mostly dunes or modern spoil from pond excavation^10^. In contrast, 56.34% of the form-line and 40% of the hachure features greater than 200 m in diameter did correspond to sites^3^. For this reason, it has been decided to filter, throughout the study area, all those mound features formed by areas of less than 500 pixels, a range of 60–150 m in diameter depending on the pixel resolution of each map, to avoid including mound features that are not likely to be archaeological sites (Filter1).

A second filter, using blob analysis, was applied to remove those elongated mound features which are not commonly archaeological sites and are mostly dunes. The ellipsoidal shape of each detected mound feature has been evaluated and all those that presented an elongation, a ratio between the largest and smallest diameter of the ellipse, greater than 3.5 were eliminated (Filter2).

**Figure 10:**
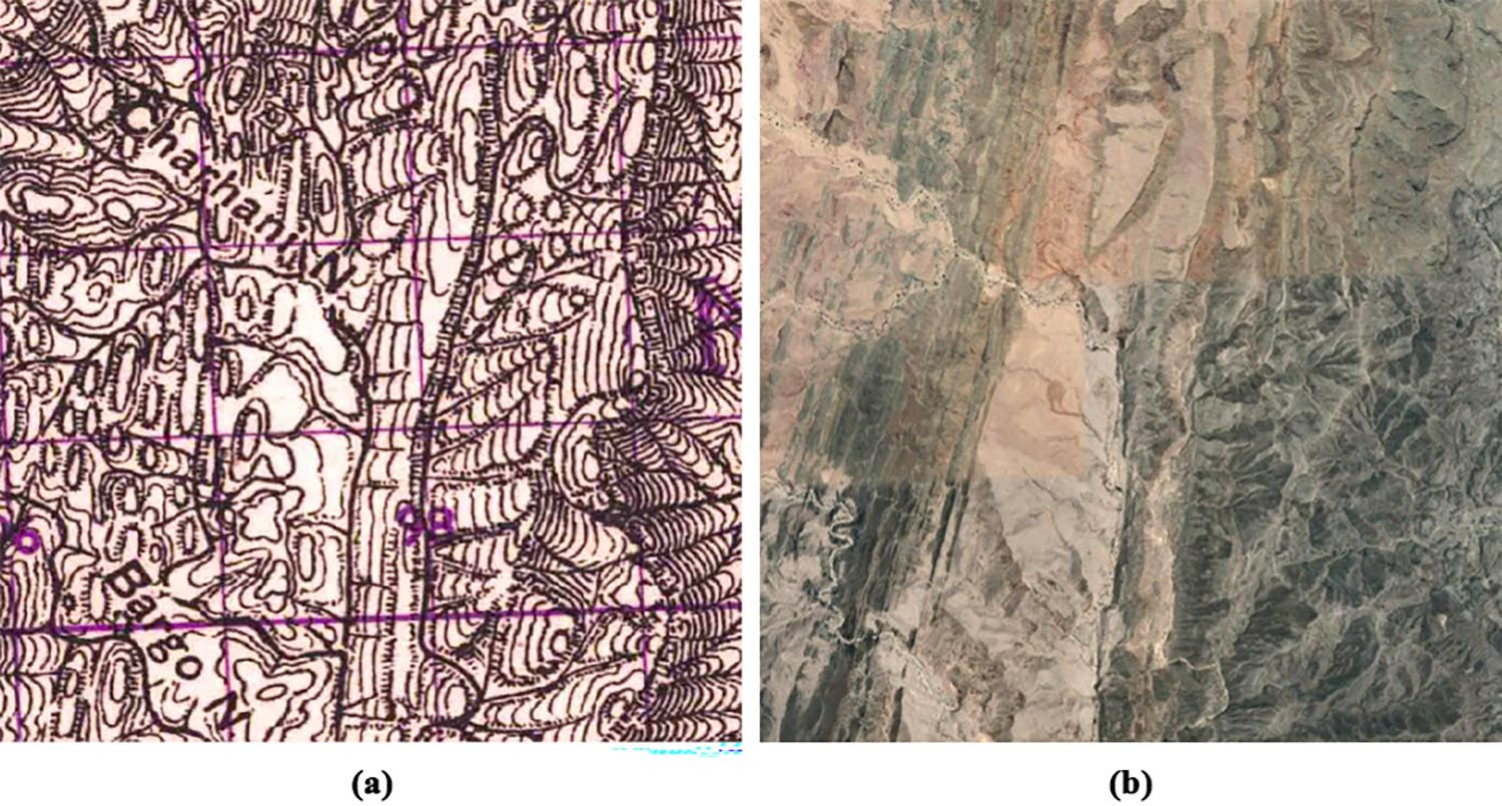
Hachure mound-shaped mountain peaks on (**a**) historical map and (**b**) its satellite image.

Finally, in the post-processing stage, given the similarity of the mound features with the characteristic elevation shape of mountainous areas, a script was applied using Google Earth Engine and QGIS to filter all those mountainous regions (Filter3), areas with a slope greater than 5 degrees (of mean value within a 7 pixel radius, equivalent in this area to 210 m), and thus eliminate all mound features that, correctly identified by their drawn shape, do not correspond to possible archaeological mounds (Fig. 10).

### Model evaluation

**Figure 11:**
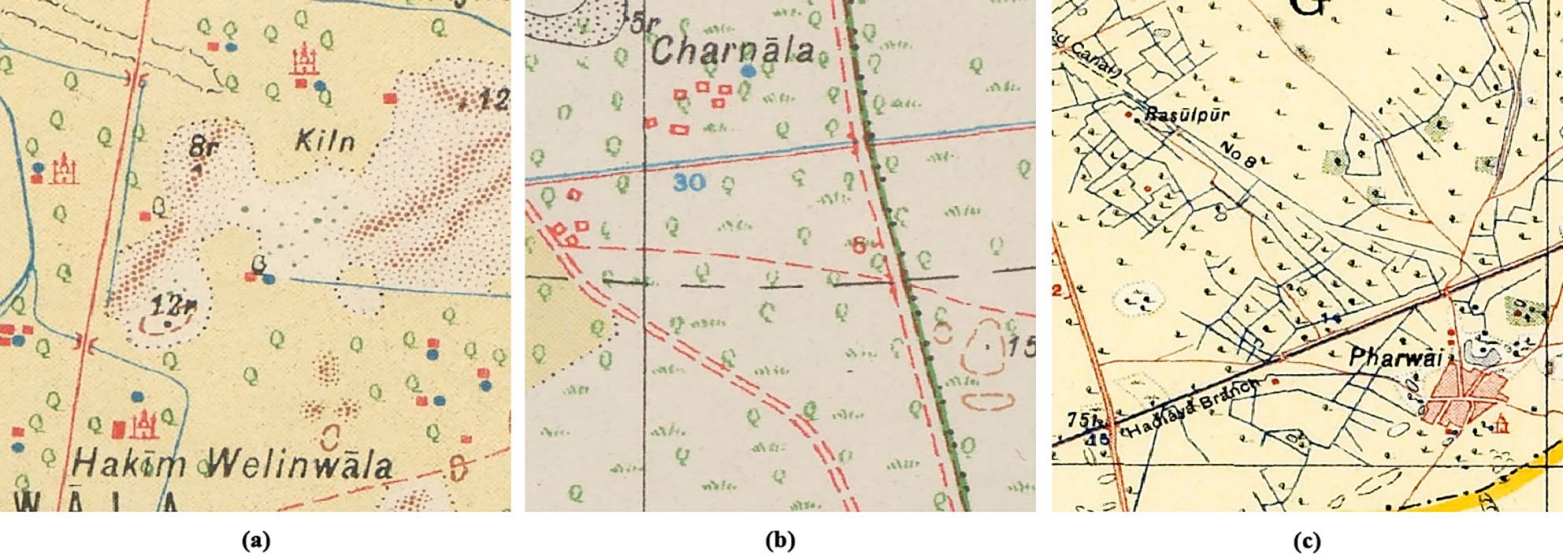
Similarity based on the RGB values of their backgrounds compared to the training and validation maps: (**a**) sample map used for training, (**b**) sample map used for test for a standard deviation of 0.5, and (**c**) sample map used for test for a standard deviation of 3.

**Figure 12:**
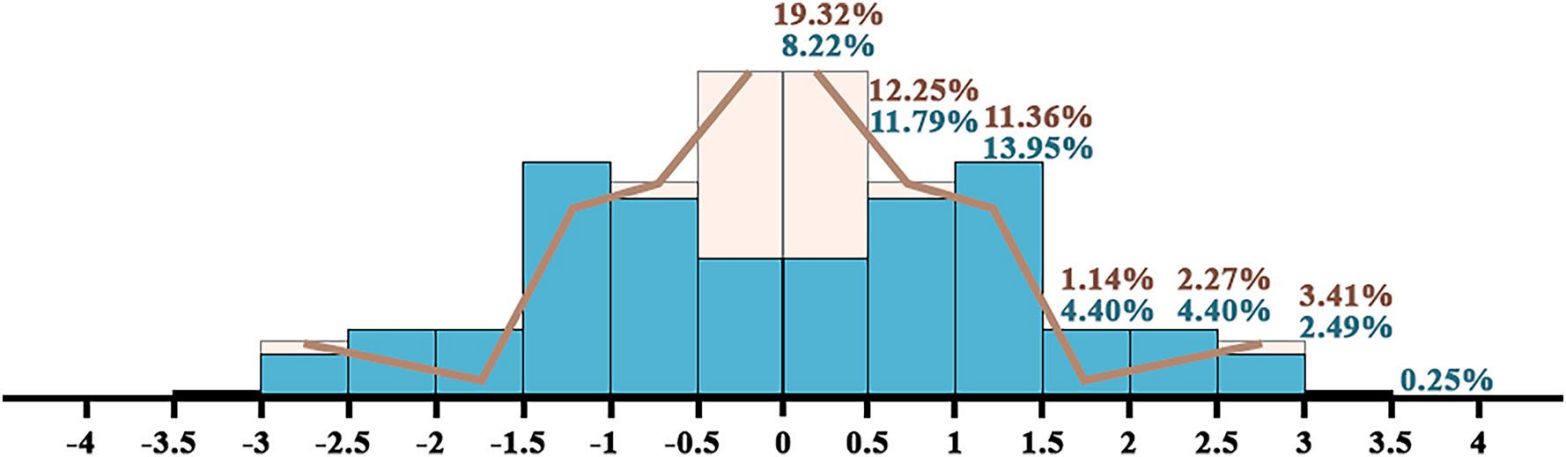
Percentage of maps in which new mounds are detected (blue) relative to the probability density of the maps used both in training and in validation (brown), their similarity based on the RGB values of their backgrounds.

Once the algorithm was trained, new mound features were detected in the remaining 581 maps for which we possessed no information on the presence of mound features. Given the diversity of the new maps compared to those used for training and validation (Fig. 11), this evaluation was carried out differentiating the maps based on their similarity with those used in training and validation following a probability density function (Fig. 12).

This detection can be replicated in Colab in order to facilitate its application by other users with the aim of making this algorithm reproducible and replicable. The resulting shapefile contains the masks of all detected mound features for easy viewing in standard GIS software such as QGIS.

## Results

Below we present the results of the workflow followed for the detection of mound features in SoI historical maps. Both the initial (Tables 1 and 2) and the final results (Tables 7 and 8) of the detection of hachure and form-line mound features are presented, and only the intermediate results of the detection of hachure as an example of the evolution of the process (Tables 3, 4, 5, and 6), which was the same for both types of mound feature representations.

**Table 1:** Evaluation of the mask R-CNN model in high and low-density validation datasets, average mound features per image, before the entire DA workflow for the detection of hachure mound features.

Algorithm	Density (%)	TPs	FNs	FPs	Recall (%)	Precision (%)	F1 (%)
High-density	128.26	87	21	26	80.56	76.99	78.73
Low-density	2.67	87	21	737	80.56	10.56	18.67

**Table 2:** Evaluation of the mask R-CNN model in high and low-density validation datasets, average mound features per image, before the entire DA workflow for the detection of form-line mound features.

Algorithm	Density (%)	TPs	FNs	FPs	Recall (%)	Precision (%)	F1 (%)
High-density	95.77	45	22	20	67.16	69.23	68.18
Low-density	1.47	45	22	1366	67.16	3.19	6.09

**Table 3:** Evaluation of the mask R-CNN models in low-density validation dataset using different DA techniques for the detection of hachure mound features: random translation (DA1), random rotation (DA2) and the so-called Doppelgänger technique (DA3).

Algorithm	TPs	FNs	FPs	Recall (%)	Precision (%)	F1 (%)
None	87	21	737	80.56	10.56	18.67
DA1	71	39	37	64.55	65.74	65.14
DA1 + DA2	68	45	53	60.18	56.20	58.12
DA1 + DA2 + DA3	68	44	31	60.71	68.69	64.45

**Table 4:** Evaluation of the Mask R-CNN models in low-density validation dataset using a refinement step (DA4) for the detection of hachure mound features.

Algorithm	TPs	FNs	FPs	Recall (%)	Precision (%)	F1 (%)
DA1 + DA2 + DA3	68	44	31	60.71	68.69	64.45
DA1 + DA2 + DA3 + DA4	70	43	19	61.95	78.65	69.31

**Table 5:** Evaluation of the Mask R-CNN models in low-density validation dataset using CL-based approach with synthetic data (DA5) for the detection of hachure mound features.

Algorithm	TPs	FNs	FPs	Recall (%)	Precision (%)	F1 (%)
DA1 + DA2 + DA3 + DA4	70	43	19	61.95	78.65	69.31
DA1 + DA2 + DA3 + DA4 + DA5	77	38	11	66.96	87.50	75.86

**Table 6:** Evaluation of area (Filter1), blob (Filter2) and slope (Filter3) filters in low-density validation dataset for the detection of hachure mound features.

Algorithm	TPs	FNs	FPs	Recall (%)	Precision (%)	F1 (%)
None	87	225	15	27.88	85.29	42.03
Filter1	78	43	13	64.46	85.71	73.58
Filter1 + Filter2	77	38	11	66.96	87.50	75.86
Filter1 + Filter2 + Filter3	77	38	10	66.96	88.51	76.24

**Table 7:** Evaluation of the mask R-CNN model in high and low-density validation datasets, average mound features per image, after the entire DA workflow for the detection of hachure mound features.

Algorithm	Density (%)	TPs	FNs	FPs	Recall (%)	Precision (%)	F1 (%)
High-density	128.26	77	38	3	66.96	96.25	78.97
Low-density	2.67	77	38	10	66.96	88.51	76.24

**Table 8:** Evaluation of the mask R-CNN model in high and low-density validation datasets, average mound features per image, after the entire DA workflow for the detection of form-line mound features.

Algorithm	Density (%)	TPs	FNs	FPs	Recall (%)	Precision (%)	F1 (%)
High-density	95.77	48	20	0	70.59	100	82.76
Low-density	1.47	48	20	4	70.59	92.31	80.00

**Figure 13:**
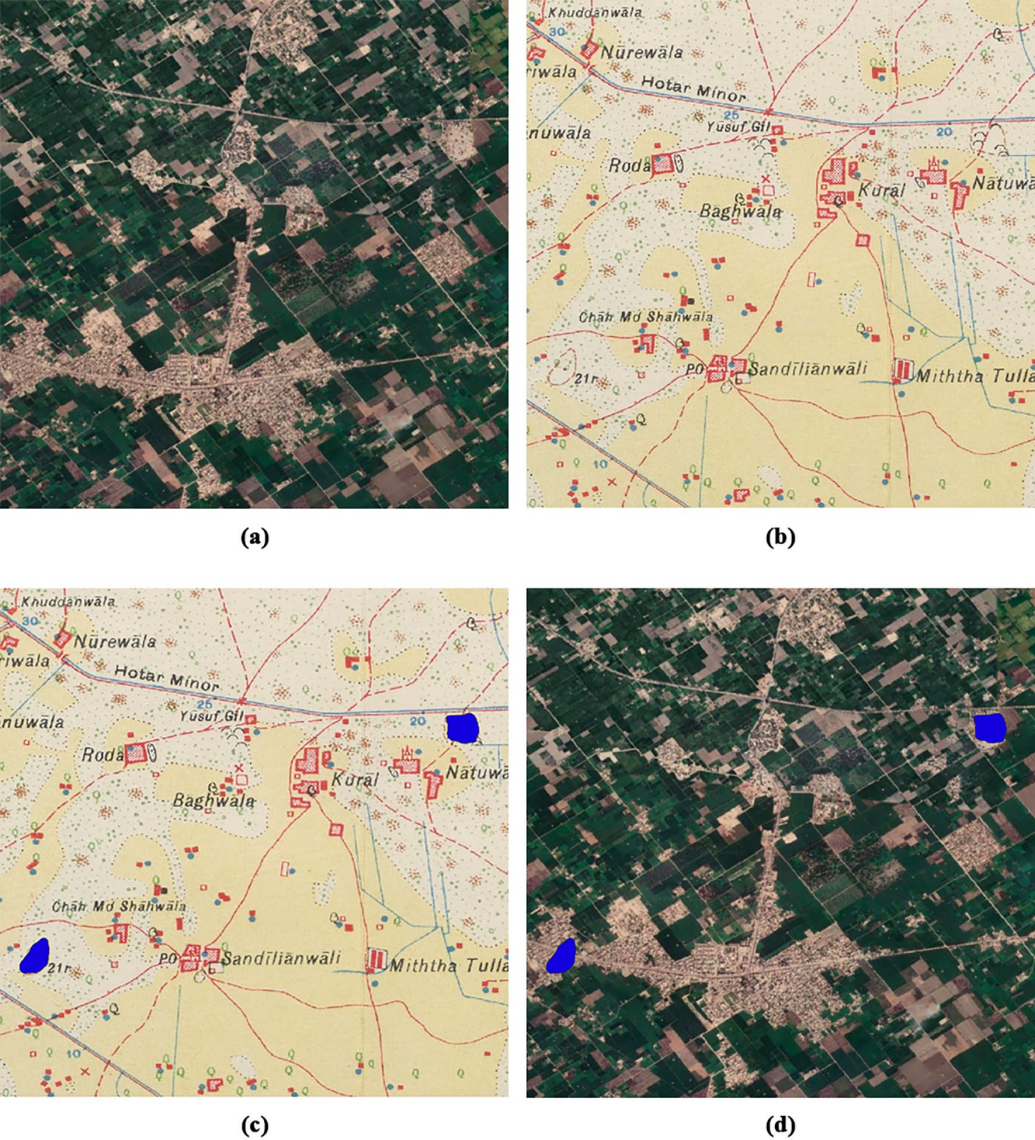
Detection of mound features [21r] in an area where urban and agricultural development have made those mapped mound features disappear: (**a**) satellite image of the area, (**b**) historical map of the area, (**c**) detection of form-line mound features (blue) on the historical map, and (**d**) location of the detected potential site mound features (blue) in the satellite image.

**Figure 14:**
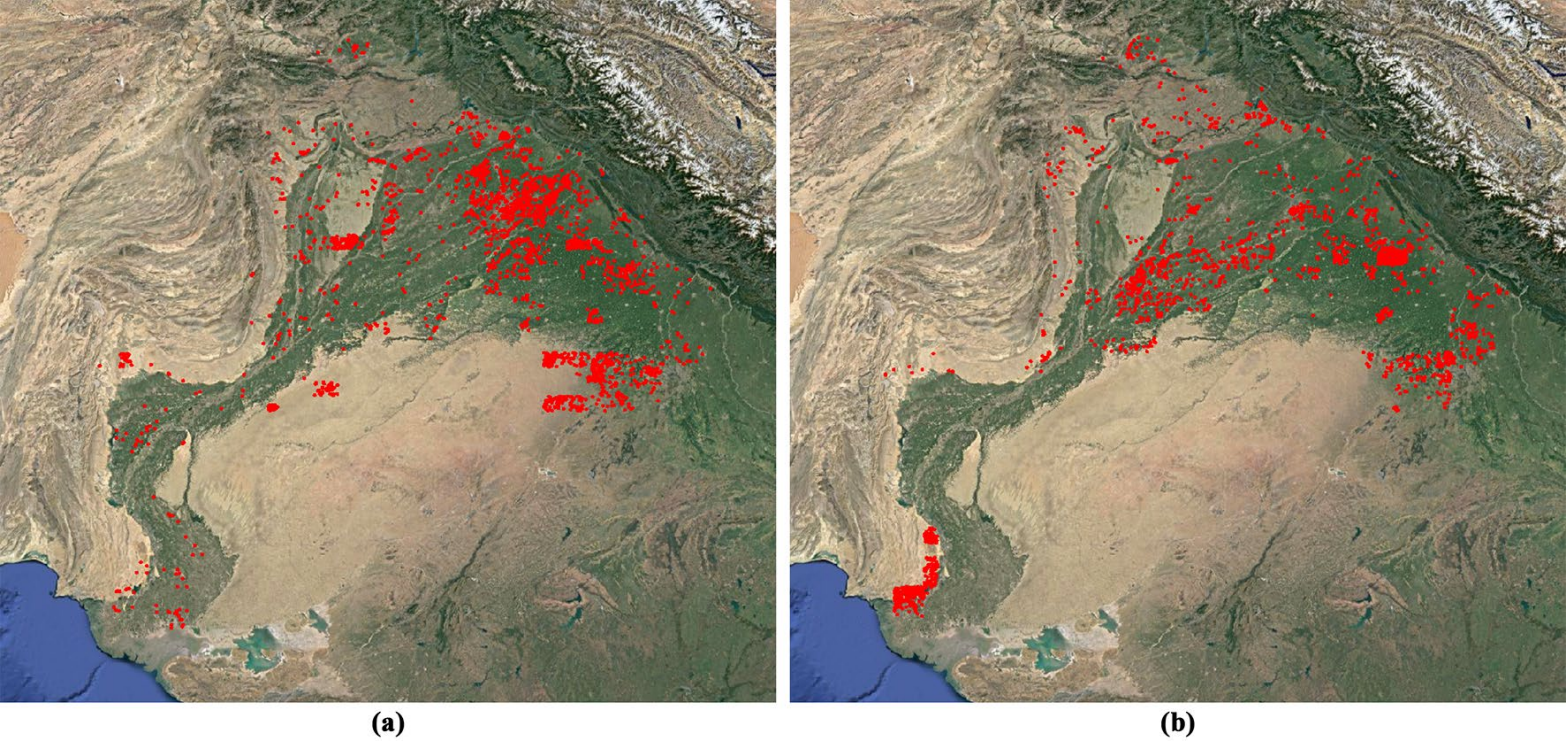
Distribution of detected mound features in the Indus River Basin: (**a**) hachure and (**b**) form-line mound features. Figure created by the first author using QGIS 3.28.4 ^13^ and a WMS-connected Google Earth satellite imagery layer as a background.

Finally, the trained model was applied to maps covering an area of 470,500 km^2^ where a total of 2802 hachure and 3145 form-line mound features have been detected (5947 mound features), and perfectly georeferenced by our algorithm (Figs. 13 and 14). A manual evaluation of a series of maps of this area was performed, the aforementioned test dataset (Tables 9 and 10).

**Table 9:** Evaluation of the mask R-CNN model in low-density test dataset based on its maps RGB similarity relative to training and validation ones for the detection of hachure mound features.

Similarity	TPs	FNs	FPs	Recall (%)	Precision (%)	F1 (%)
|0.5σ|	92	61	9	60.13	91.09	72.44
|1σ|	111	89	14	55.50	88.80	68.31
|2σ|	116	104	19	52.73	85.93	65.35
|3σ|	121	109	26	52.61	82.31	64.19

**Table 10:** Evaluation of the Mask R-CNN model in low-density test dataset based on its maps RGB similarity relative to training and validation ones for the detection of form-line mound features.

Similarity	TPs	FNs	FPs	Recall (%)	Precision (%)	F1 (%)
|0.5σ|	15	1	1	93.75	93.75	93.75
|1σ|	25	6	5*	80.65	83.33	81.97
|2σ|	97	40	27	70.80	78.23	74.33
|3σ|	97	40	41	70.80	70.29	70.55

*Four of the detected mound features were drawn in another way than the one used for training, the continuous form-line. For this reason, they have not been taken into account either as TP or as FP.

## Discussion

### Low-density approach

In archaeology, it is common to find unsatisfactory results masked by the difference in the density of archaeological features. The density of the features must be taken into account^11,12^ since good results in high-density areas may actually be hiding much worse results in low-density areas. The first results showed a number of FPs of up to twenty times more than the mound features present in the area (Tables 1 and 2). This algorithm would be useless in a large-scale survey, as it would generate a large number of FPs and an overly large dataset, which would not be of use in the planning of field validation or for archaeological analysis. These results strongly show that archaeological studies should focus their validation on low-density areas in order to avoid biased results.

During an archaeological survey, the true density of archaeological features is unknown, so algorithms must be developed to show good metrics in areas of both high and low-density of sites. Contrary to recently published discussions^12^, poor results in low-density areas due to the sparse presence of archaeological features and class imbalance are not inevitable, but these are the product of insufficient model training. The foreground-to-background imbalance as an example of class imbalance^22^, is not the reason for poor results in the detection stage. The imbalance problem from each category for object detection in the training pipeline^23^, occurs when one class heavily outnumbers the examples in the other class in the training data^24^, not in the validation and test datasets. Variation in results due to the different density of archaeological features (Tables 1 and 2) can be resolved by different DA and CL approaches (Tables 7 and 8).

### Model refinement and curriculum learning approach

The DA, with the introduction of 1500 new mound features, significantly improves the precision by increasing the training data, both positive and negative. Both DA1 and DA2 show similar results that, despite the slight reduction in recall we have achieved a substantial improvement in precision (Table 3). Thanks to its negative training, the introduction of DA3 improves the precision of the model, which uses the DA4 to improve its accuracy.

The initial training data was not sufficient and resulted in a large number of FPs indicating that the model had not learned well what a mound feature looks like. The increase of the training data removed a large number of FPs, but to eliminate more specific FPs it was necessary to resort to DA3 and DA4 (Table 8). As shown in Fig. 7, most of the FPs used in refinement were pointed circular and non-circular shapes for the hachure algorithm, and both continuous and dashed circular shapes for the form-line model.

**Figure 15:**
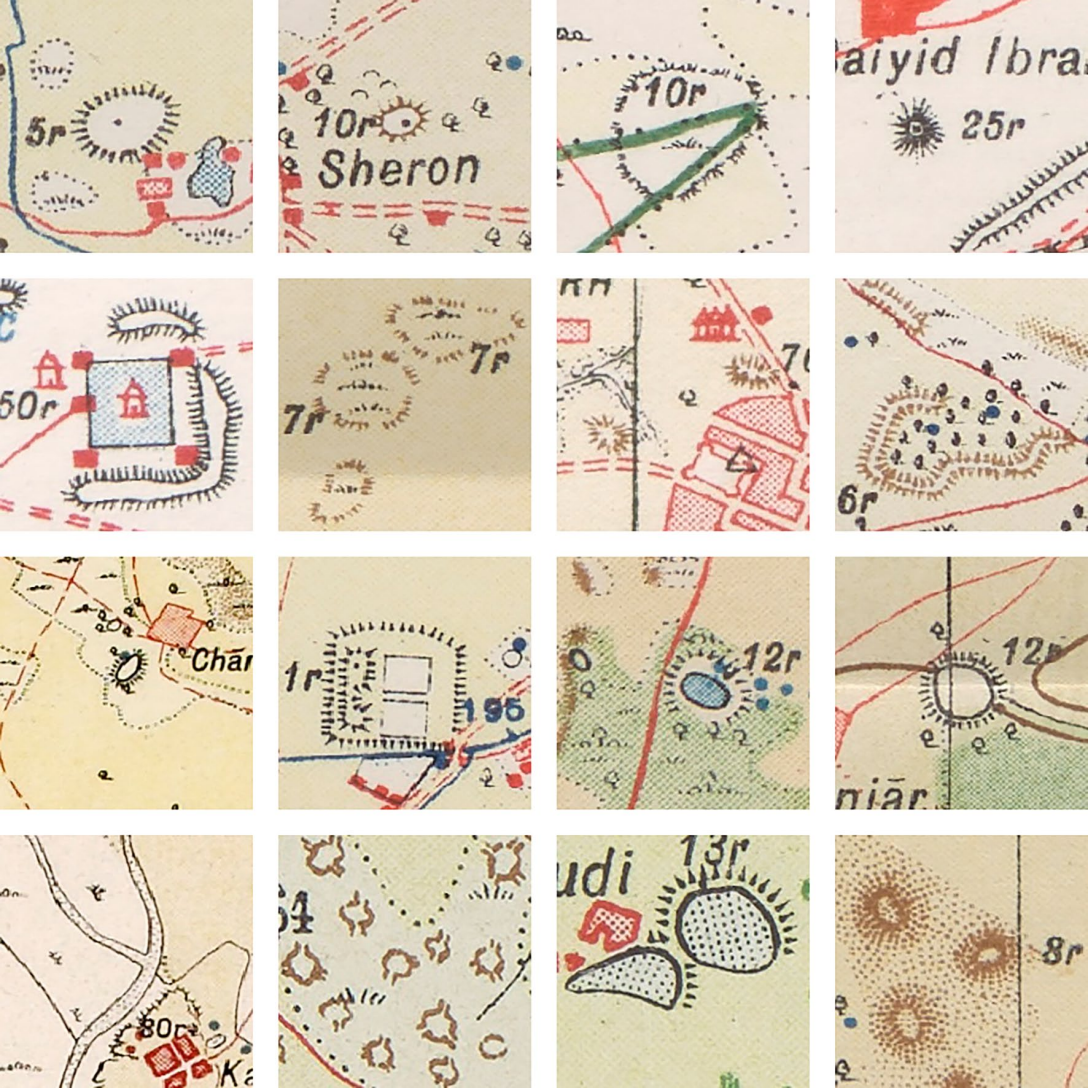
Different types of hachure mound features detected after applying the trained model. The last image represents the third type of mound features on the maps, the shaded relief mound features, erroneously detected as hachure but similar to them due to their characteristic pointed and circular shapes.

Likewise, as can be seen in Fig. 15, the use of DA5 has allowed the detection of hachure shapes not included in the original training data. The inclusion of synthetic data, along with the CL strategy, has allowed the algorithm to better understand what the mound features look like. The CL using synthetic data helped to develop an algorithm from a small training dataset, which is common in archaeology. As seen in Table 5, both the recall value and the precision value improved noticeably.

### Model filtering

Smaller objects, such as mound features less than 500 pixels in area, are the most difficult for a CNN to detect, because such objects do not have enough pixels for the necessary feature extraction. That is why the recall value is so low without Filter1 but high enough when we apply it (Table 6). Both Filter2 and Filter3 remove many FPs, which results in an increase of the precision of the model, with fewer, but higher quality results that are more likely to be of archaeological interest.

In future work, the idea of developing new filters could be contemplated for the elimination of mound features correctly detected but not correctly classified in their type. Some hachure mound features, in addition to being detected by the hachure algorithm, have been detected by the form-line mound features algorithm. What has been detected is not the complete mound feature but only its interior, which on many occasions resembles a form-line mound feature. These misclassified mound features could easily be removed with a filter that discards the smallest duplicate detected mound feature. This can also happen with the hachure and the shaded-relief mound features. Some shaded-relief examples, as the last image of Fig. 15, resembles a hachure mound feature. Applying the same filter mentioned above would also resolve these double detections, as well as reduce the FPs for shaded-like dunes.

### Model evaluation

Only 40.03% of the maps with unknown mound features, the ones used for testing, are similar to 63.64% of the maps used for training and validation (Fig. 12), so most are substantially different. This diversity as well as its resulting metrics (Tables 9 and 10) indicate the need for an adaptive algorithm that allows, after a small amount of retraining with data detected from a new map, a better general mound feature detection in the same map. The more similar the maps are to those used in training and validation, the more similar the test metrics are to the validation ones. An adaptive algorithm would improve both recall value by including different ways of drawing the mound features, only some of which have been detected thanks to the synthetic data, and precision value by including backgrounds not taken into account in the original training.

**Figure 16:**
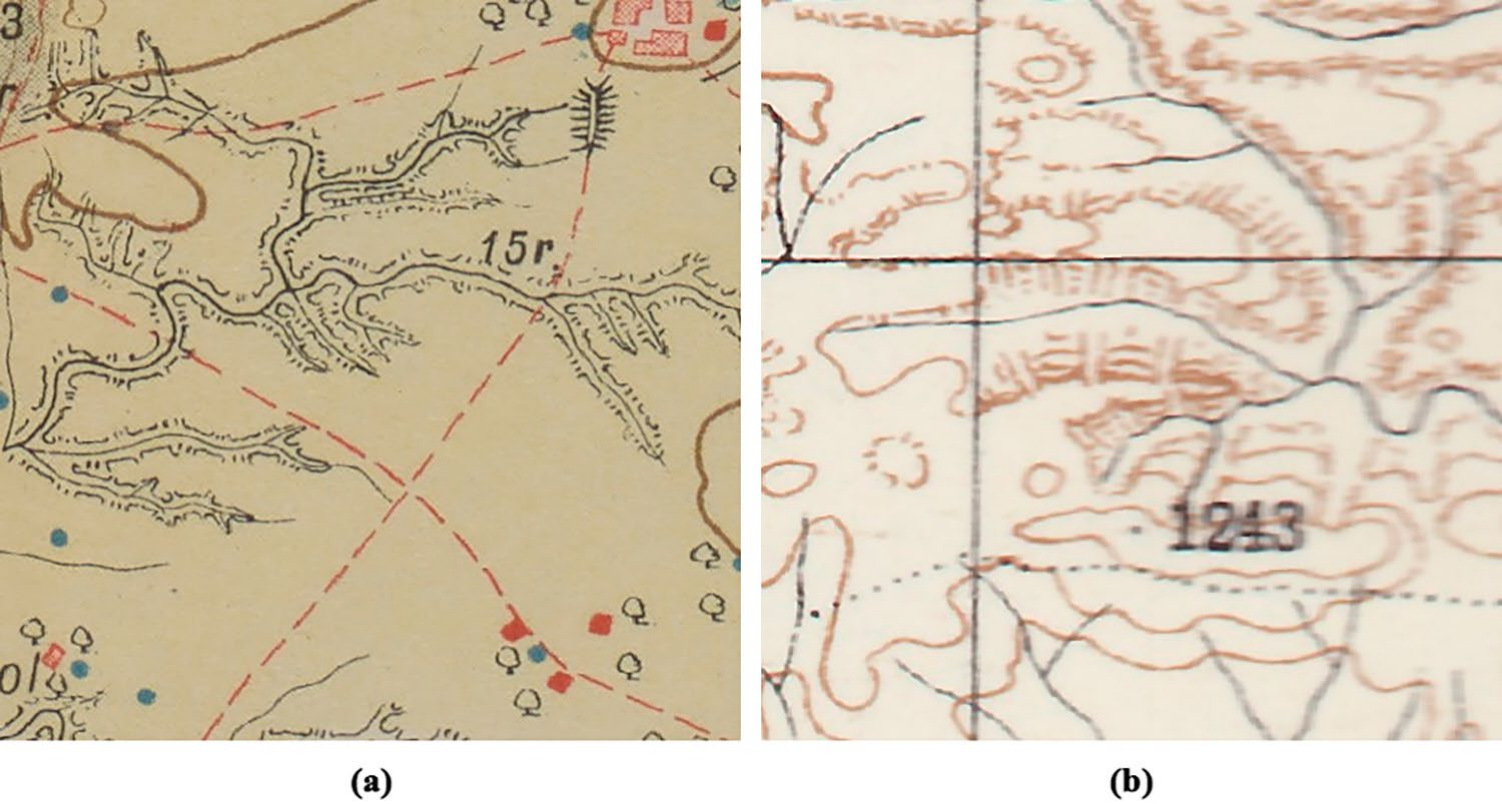
Map samples found in the test data with different characteristics than those used in training and validation: (**a**) darker image background and (**b**) blurred image.

Likewise, new DA methods could be included in the training, such as random brightness jittering and random Blur/Sharpen^25^. Some test maps, unlike those used in training and validation, have shown darker and blurred images (Fig. 16).

### Comparison to manual digitisation of mound features

The VIA annotation software was used to hand digitise 756 mound features in JSON format, which were digitised using 64 random historical maps. The density of mound features is not distributed uniformly throughout each map. Instead, mound features frequently cluster together, indicating a high number of mound features on certain maps and a low number on others. This type of pattern increases the amount of labour and time necessary for manual mound feature digitising using GIS software. We predicted that manually digitising all mound features from the 645 historical maps used in this research region would take an experienced professional more than 120 work hours based on the manually digitised mound features prepared as training data for the algorithm. The detection time, running each algorithm on a single NVIDIA A40 GPU, has been more than 6 computing hours. While 120 h does not seem too long for this project, creating a ML-based algorithm paves the way to scale this research to the additional 2200 historical maps covering other parts of Pakistan and India that have been scanned and are ready for analysis.

## Conclusions

A workflow has been designed with different techniques and strategies that has allowed not only the detection of nearly 6000 mound features in India and Pakistan, which will allow for a better understanding of the settlement distributions related to the Indus Civilization and later cultural periods, but has also provided solutions to common problems in archaeology such as the low-density of archaeological features in large-scale surveys and the few training data for ML models.

Historical maps constitute one of the basic sources available to both historians and archaeologists. The study area analysed in this paper present an excellent case. Much of the information provided by the maps cannot be obtained using other survey methods as the area has been systematically modified during the last century. This is also the case of many other areas where systematic landscape modifications have been implemented and for which historical map series exist^26^. These are housed in many archives and some series cover very large national and colonial territories using very similar symbols and conventions. This study opens the door for the large-scale automated extraction of relevant information from historical maps and, in doing so, provides a workflow and open code that has the potential to immensely contribute to the historical sciences.

As with other large-scale site detection methods^4^, these DL algorithms will allow researchers to carry out studies that could not be done before given the new amount of data obtained, facilitating the task of the archaeologist. Furthermore, this model could be applied in other regions that have historical maps such as Syria and Lebanon^9^, but particularly those areas that were also mapped by or followed the model established by the SoI. The outputs of this study represent a powerful tool in the large-scale documentation and monitoring of archaeological heritage, with much work ahead to validate the results through remote sensing, archival work, and ground survey in collaboration with partners in India and Pakistan.

## Data Availability

The historical map datasets generated and/or analysed during the current study are scheduled to be made publicly available via the British Library and Cambridge University Library digital data repositories. Until that occurs, they are available from the corresponding author on reasonable request. The historical map mound feature dataset generated and/or analysed during the current study are scheduled to be made publicly available via the Arches instance hosted by the Mapping Archaeological Heritage in South Asia (MAHSA) project. Until that occurs, they are available from the corresponding author on reasonable request. The supplementary code for the Data Augmentation process can be found online at https://github.com/iberganzo/ArchaeolDA.
